# 不可切除气管、支气管内肿瘤的纤维支气管镜CO_2_冷冻治疗

**DOI:** 10.3779/j.issn.1009-3419.2014.07.08

**Published:** 2014-07-20

**Authors:** 千里 马, 彬 石, 燕雏 田, 德若 刘

**Affiliations:** 100029 北京，中日友好医院胸外科 Department of General Thoracic Surgery, China-Japan Friendship Hospital, Beijing 100029, China

**Keywords:** 纤维支气管镜, 外科冷冻, 气管、支气管肿瘤, Bronchoscopy, Cryosurgery, Endobronchial tumor

## Abstract

**背景与目的:**

气管、支气管肿瘤性的狭窄或闭塞，大多发生于病程的晚期，患者会出现十分痛苦的症状，如呼吸困难、咯血、阻塞性肺炎和顽固性高热。全身情况较差，心肺功能受限，原发灶和转移灶均不能耐受根治性手术切除。呼吸道梗阻严重影响了生存质量，甚至引起呼吸衰竭导致死亡。因此，一种能够有效解除梗阻的治疗方法就显得尤为重要。我们选择在纤维支气管镜下运用二氧化碳（carbon dioxide, CO_2_）冷冻技术对气管、支气管腔内肿瘤进行切除，探讨其治疗效果。

**方法:**

对126例气管、支气管肿瘤进行冷冻治疗（2004年8月-2014年2月），观察近期及远期治疗效果。术前需要进行计算机断层扫描（computed tomography, CT）加气管、支气管的三维重建，纤维气管镜检查后评估冷冻的部位和深度；术中采用全麻或表面麻醉加静脉强化，置入纤维支气管镜至肿物上端约0.5 cm，后将软式冷冻探头经支气管镜活检孔插入。冷冻探头的金属末端置于肿瘤中心，冷冻约30 s-120 s，冷冻温度-50 ℃--70 ℃。在冷冻后未完全溶解前“撕脱”切除肿瘤（冻切法）或者冻时间4 min-6 min后通过负压吸引清除冷冻后坏死的肿瘤组织（冻融法），两种方法相结合可以较为彻底地清除气道内肿瘤，直至管腔通畅。2周后复查气管镜，决定是否需要再次冷冻治疗。

**结果:**

患者咳嗽、呼吸困难、咯血均有不同程度缓解，显效率为65.1%，总有效率77.0%。围手术期死亡1例，术后气道内出血2例，支气管瘢痕狭窄4例，气管烧灼伤2例，气管软化2例，心房纤颤3例。中位生存期为14个月，1年、2年、3年的生存率分别为58.6%、24.2%、12.2%。

**结论:**

纤维支气管镜CO_2_冷冻技术是一种十分简便而有效的微创治疗方法。治疗管腔内生长的中央型气道肿瘤，可以去除肿瘤阻塞气道的部分，重新疏通管腔，迅速控制和缓解气道梗阻所导致的症状，显著提高生存质量。部分患者可以解决麻醉气管插管问题，为下一步治疗创造条件，从而达到根治性切除原发肿瘤的目的。

## Abstract (en)

**Background and objective:**

Patients with tracheobronchial tumor are too frail for curative surgical treatment, and the original sites are unsuitable for radical resection. They always suffered from dyspnea, hymoptosis, obstructive pneumonia, and fever. Airway obstruction has a strong bad impact on the quality of life, and is the main cause of respiratory failure and death. An effective method of palliative is very important. We choose fibro-bronchoscopic cryosurgery for destruction of endobronchial tumors and analyze the outcomes.

**Methods:**

Clinical records of 126 cases who under went endobronchoscopic cryosurgery were reviewed retrospectively from August 2004 to February 2014. Techniques and survival outcomes were analyzed. The precise position and length of the cryosurgery was evaluated through three dimension chest computed tomography (CT) and reconstruction of trachea and bronchus. Local infiltration and general intravenous anesthesia are two options before surgical procedure. Endobronchial tumor was removed by combining two methods of "Cryo-Resecion" and "Cryo-Melt". Bronchoscopic examination was rechecked 2 weeks later to evaluate if it is necessary to redo the endobronchoscopic cryosurgery.

**Results:**

The symptoms of cough, short of breath, and hemoptysis were alleviated to different extend. The rate of dramatically improved and partial improved were 65.1% and 77.0% respectively. The mortality is 0.79%. Postoperative Complications included 2 cases of airway bleeding, 4 cases of bronchial stenosis, 2 cases of trachea burning injury, 2 cases of tracheomalacia, and 3 cases of atrium fibrillation. The median survival is 14 months, 1-, 2-, 3-yr survival rates were 58.6%, 24.2%, and 12.2% respectively, based on *Kaplan*-*Meier* estimates.

**Conclusion:**

Endobronchial cryosurgery is an easy and effective minimally invasive choice for controlling and releasing the airway obstructive symptoms resulting from the trachea or bronchial tumors. Besides, the problem of difficult endotracheal intubation could be resolved after removing the tracheal tumor with the benefit of cryotreatment. Some patients could get the chance for further treatment or radical resection. Therefore, the patients' quality of life could be improved and the lifetime could be prolonged.

气管、支气管肿瘤患者在确诊时大多处于中晚期，往往会出现十分痛苦的症状，如呼吸困难、咯血、阻塞性肺炎和顽固性高热。为改善患者的生活质量，延长生存期，目前用于治疗气道内肿瘤的技术包括：外部光束放射治疗法（external beam radiation, EBR）、钇铝石榴石晶体（neodymium-doped yttrium aluminium garnet, ND: YAG）激光疗法和气管内放射治疗。由于硬式支气管镜在适应证和操作上存在一定的局限性和危险性，所以我院与北京库兰医疗设备有限公司合作，开发出超细的可弯曲的软式冷冻探头（图 1），可以在纤维支气管镜下完成气道内肿瘤CO_2_的冷冻切除手术。我院从2004年8月-2014年2月治疗气道内肿瘤126例，取得了较好的效果。

**1:**
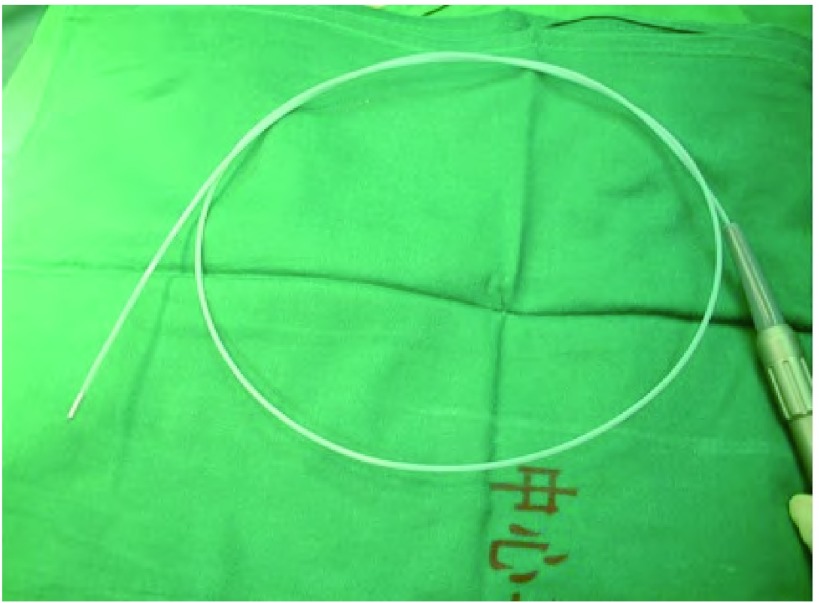
超细的可弯曲的软式冷冻探头 Superfine curved cryosurgical probe

## 1 资料与方法

### 1.1 一般资料

共126例患者，男性98例，女性28例。年龄21岁-80岁，平均年龄64岁。其中鳞癌69例，腺癌13例，腺样囊性癌16例，支气管内肉芽肿8例，支气管淀粉样变2例，癌肉瘤4例，小细胞肺癌5例，食管癌转移5例，甲状腺癌直接侵犯2例，腹壁平滑肌肉瘤转移1例，结肠癌转移1例。病变的部位：气管15例，左主支气管28例，左肺上叶支气管14例，左肺下叶支气管12例，右主支气管32例，右肺上叶支气管13例，右肺下叶支气管12例（表 1）。126例均有不同程度的咳嗽、咯血、呼吸困难、阻塞性肺炎或顽固性高热。属于晚期或术后复发，或心肺功能受限不能耐受手术，或者拒绝手术治疗。

**1:** 临床特点 Clinical characteristics

Characteristic		*n* (%)
Gender	Male	98 (77.8)
	Female	28 (22.2)
Age		62±7
Pathology	Squamous carcinoma	69 (54.8)
	Adenocaicinoma	13 (10.3)
	Adenoid cystic carcinoma	16 (12.7)
	Granulomatous	8 (6.3)
	Amyloidosis	2 (1.6)
	Sarcoma	4 (3.2)
	Small cell carcinoma	5 (4.0)
	Esophageal carcinoma	5 (4.0)
	Thyroid carcinoma	2 (1.6)
	Leiomyosarcoma	1 (0.8)
	Colon cancer	1 (0.8)
Position	Trachea	15 (11.9)
	Left main bronchus	28 (22.2)
	Left upper bronchus	14 (11.1)
	Left lower bronchus	12 (9.5)
	Right main bronchus	32 (25.4)
	Right upper bronchus	13 (10.3)
	Right lower bronchus	12 (9.5)
Stage	Ⅱ	9 (7.1)
	Ⅲa	28 (22.2)
	Ⅲb	18 (14.3)
	Ⅳ	53 (42.1)
	Undefined	18 (14.3)
Other treatments	Postoperative radiation	51 (40.5)
	Preoperative radiation	13 (10.3)
	Postoperative chemotherapy	8 (6.3)
	Preoperative chemotherapy	23 (18.3)
	Lung resection	7 (5.6)
Complications	Bleeding	2 (1.6)
	Atrial fibrillation	3 (2.4)
	Airway burning injury	2 (1.6)
	Tracheal stenosis	4 (3.2)
	Tracheal malacia	2 (1.6)
	Respiratory failure	2 (1.6)

### 1.2 材料和器械

冷冻治疗仪和软式可弯曲冷冻探头Kooland 300和Kooland 320（北京库兰医疗设备有限公司），探头直径1.2 mm-2.0 mm，长度90 cm，探头末端长度5 mm。冷源为液态CO_2_。内镜工作通道大于2.0 mm的Olympus BF-IT20或者BF-260电子纤维支气管镜。

### 1.3 患者的准备

查胸部CT加气管、支气管三围重建，出凝血时间，术前做纤维或电子支气管镜了解肿瘤的大小、性质、部位和范围，同时还应对患者的肺功能、动脉血气分析，评估肺不张的时间。

### 1.4 术前准备

术前6 h禁食水，采用全身麻醉或者用4%丁卡因鼻腔及咽喉部喷雾粘膜表面麻醉，插入纤维支气管镜后经活检孔注入2%利多卡因5 mL-20 mL，以麻醉咽后壁、声带及气管。用75%酒精对冷冻探头进行消毒。

### 1.5 操作步骤

麻醉采用全身麻醉或者表面辅加静脉强化麻醉。气管镜对气管各段支气管检查后明确病变部位、大小、阻塞的程度，决定冷冻的方法和持续的时间。病灶表面有坏死物或痰液时予以清理，以使探头与病灶有充分的接触。治疗时，将纤维支气管镜置入气道内直至肿瘤上方0.5 cm处，而后将冷冻探头经支气管镜的活检口插入，进而置入肿瘤的中心，可采用垂直或者切线方向对肿瘤进行冷冻。具体的处理方法为首先进行“冻融法”：踩下踏板开始对探头尖端进行冷却，约15 s-30 s后形成一个冰球，温度可达-50℃--70℃，持续时间30 s-4 min。肉眼可见组织发白、局部形成冰晶，松开脚踏板后让其自然融化，称之为一个冷冻-消融循环。根据病变的大小可以在肿瘤不同的区域设定冷冻点，每个冷冻点进行1个-3个循环，最终将肿瘤气道内可见部分完全冷冻。之后采用“冻切法”：待探头尖端与肿瘤切线方向产生最大的冷冻效果后，缓慢地拔出探头，将冷冻的部分肿瘤组织从瘤体上撕脱下来，反复多次操作，直到管腔内全部肿瘤或者大部分肿瘤切除。治疗后吸出气道内分泌物、脱落坏死的组织及渗血。仔细检查操作的创面，可以采用1:1, 000的肾上腺素或者立止血1 ku对局部喷洒止血。出院2周后复查气管镜，对疗效进行评价，若阻塞仍然存在或者效果不理想时可以反复多次处理。本中心冷冻处理次数为1次-8次，平均2.3次。

### 1.6 疗效判定

无论是原发气管肿瘤还是转移性气管肿瘤，在不能外科治疗的情况下，纤维支气管镜CO_2_冷冻技术能明显减轻梗阻症状，尤其是恶性肿瘤的姑息性切除能够明显解除气道梗阻，提高肺功能。由于一次治疗有导致气道壁穿孔或者大出血的可能，可以根据患者的临床情况决定多次处理。通过胸部CT或者内镜观察，对比治疗前后病灶的大小、气道狭窄的程度及咯血、呼吸困难等主要症状缓解的情况，作为疗效判断的依据。治疗效果分为三个级别：①显效：1次-3次后咳嗽、呼吸困难、咯血、胸痛明显减轻，胸部影像学示气道拓宽，内镜下肿块完全切除或者缩小2/3，狭窄的气道完全打通或明显增宽（图 2）；②有效：治疗后临床表现改善，内镜下肿块缩小1/3，狭窄的气道略有变宽；③无效：临床表现无好转，内镜下肿块大小、气道直径没有变化。统计所用软件为SPSS 20.0。

**2:**
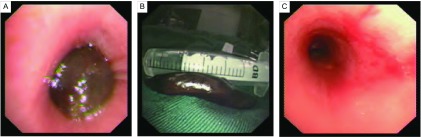
左主支气管内平滑肌肉瘤。A：气管镜下可见左主支气管完全被一肿物阻塞，表面光滑，质地柔软；B：联合“冻切法”及“冻融法”处理肿物（1.5 cm×5 cm），并将其完全取出；C：肿瘤取出后，左主支气管官腔通畅，蒂位于内侧壁，氩气刀烧灼基底部。 Cryosurgery for the left main bronchus leiomyosarcoma. A: The left main bronchus was totally obstructed by a tumor. The tumor was smooth and soft; B: Two methods of "Cryo-Resection" and "Cryo-Melt" were combined to remove the tumor totally (1.5 cm×5 cm); C: The left main bronchus was unobstructed after removing the tumor. The pedicel of the tumor was located on the bronchial internal wall, and the basement was cauterized by argon knife.

## 2 结果

### 2.1 术后肿块大小、临床表现的改善情况

本中心冷冻后患者自觉症状均有不同程度的改善，其中喘息、呼吸困难改善率分别为79.8%（91/114），咳嗽75.9%（82/108），咯血37.0%（27/73），胸痛64.6%（53/82），发热63.9%（23/36）（表 2）。初次治疗后即刻气道出现通畅者53例，经过2次-5次治疗后缓解者29例。显效率为65.1%，总有效率77.0%，无效12例。冷冻术后放疗可以显著提高局部控制率。中位生存期为14个月，1年、2年、3年的生存率分别为58.6%、24.2%、12.2%（图 3）。

**2:** 冷冻治疗后症状缓解情况 Extend of the clinical symptoms alleviation after endobronchial cryosurgery

Symptoms	Before treatment	After treatment			Ratio of effectivenes
		Dramatically improved	Improved	No effect	
Dyspnea	114	61	30	23	79.8%
Cough	108	50	32	26	75.9%
Hemoptosis	73	17	10	46	37.0%
Fever	36	17	6	13	63.9%
Chest pain	82	21	32	29	64.6%

**3:**
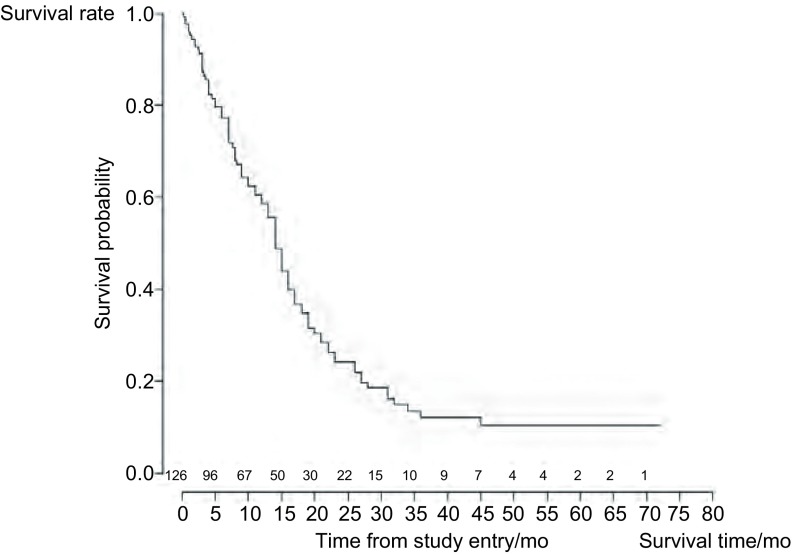
气管、支气管内肿瘤患者纤维支气管镜CO_2_冷冻术后长期生存曲线 Long term survival curve for patients with tracheal-bronchial tumor after fibrobronscopic CO_2_ cryosurgery

### 2.2 并发症与不良反应

患者均可以耐受，无气道穿孔，围手术期死亡1例，术后气道内出血2例，均再次气管插管接呼吸机辅助呼吸，4例出现气道瘢痕狭窄，2例在用氩气刀止血时出现气管灼伤，2例出现气管软化行支架处理，3例出现心房纤颤给予对症处理后好转。

## 3 讨论

几个世纪以来人们就知道冰具有镇静、止血、止痛和抗炎的特点。1899年，White在纽约第1次报道成功应用液态氧治疗红斑狼疮、带状疱疹等^[1]^。1935年，美国芝加哥的Pusey第1次报道用CO_2_治疗黑痔^[2]^。1961年美国外科医生Cooper采用了封闭的液氮冷冻探头对很多部位的癌症进行局部低温冷冻治疗^[3]^。1999年英国学者Maiwand报道用冷冻姑息性治疗气管内肿瘤153例，取得了成功的经验^[4]^。

不同组织对冷冻的敏感程度不同，通常含水多的组织（如皮肤、粘膜、肉芽组织等）对冷冻相比较敏感，而一些含水较少的组织（如脂肪、骨骼、纤维结缔组织等）对冷冻的耐受性较好，一般来说，肿瘤细胞要比普通细胞对冷冻更加敏感^[5]^。应用冷冻可使肿瘤组织凝固，结成冰块，继而微血栓形成，导致选择性的细胞坏死，减少肿瘤的扩散。同时使组织粗糙，产生粘连，避免肿瘤坏死后继发性出血。另外，冷冻可以引起炎症反应，大量白细胞浸润，增强局部免疫作用，冷冻后原位瘤可产生该肿瘤的特异性移植抗原（tumor specific transplantation antigens, TSTA），并刺激机体产生特异性抗体，达到排斥肿瘤的特异免疫效果^[6]^。研究^[7]^发现冷冻治疗辅助放、化疗会更有效，这对控制肿瘤生长、延长患者生存生存时间具有重要的作用。

中日友好医院胸外科在20世纪80年代开始进行冷冻治疗手术，并在2002年率先在国内开展了硬式气管镜下冷冻治疗，取得了较好的效果^[5]^。从2003年开始，与北京库兰医疗设备有限公司合作研制出纤维支气管镜专用冷冻探头，应用于临床后取得了良好的治疗效果。大部分患者的临床症状得以缓解，生活质量得以提高，中位生存期达到14个月，3年的生存率为12.2%。晚期肿瘤导致大气道堵塞是非常棘手的一个问题，与激光和电凝相比，冷冻具有费用低，易防护，不易发生气管、支气管壁穿孔和腔内燃烧的危险。本中心在治疗过程中出现的最大风险是气道内出血，采用在气管镜引导下将气管插管插到出血对侧的主支气管保证健侧通气，同时利用气管插管的套囊压迫出血部位可以很好的起到止血作用。另外，比较凶险的是操作过程中出现气管烧伤，这2例灼伤的患者均在操作初期出现。总结经验教训后，在用氩气刀止血时，停止供氧，采取“点烧”止血，而非长时间的按压烧灼等预防措施后，未再出现气管灼伤的情况。

硬式气管镜及硬式探头需要全身麻醉和高频通气，上叶远端的病变无法到达，与之相比，细小可弯曲的柔软纤维冷冻探头是低温疗法在内镜下治疗气道内病变的一个革新，可以清除所用气道内部的肿瘤组织，避免了因全麻及硬式支气管镜所带来的危险。虽然从整体上来说，冷冻治疗仅能清除镜下所见部分，治疗并非根治性切除，术后复发或者转移率仍然较高，但是对于存在明显气道腔内病变的患者来说，大多数患者在接受冷冻治疗后，气道堵塞的症状可以得到明显改善，大大提高了生活质量。

纤维支气管镜CO_2_冷冻治疗气管、支气管内肿瘤可以在局麻下进行，安全可靠，是一种十分简便而有效的微创治疗方法。可以迅速控制和缓解气道梗阻症状和改善生存质量，为下一步治疗创造条件；部分患者可以解决麻醉气管插管问题，从而获得根治性切除原发灶和侵犯部位的机会，达到治愈的目的。
